# Bilingual Advantages in Inhibition or Selective Attention: More Challenges

**DOI:** 10.3389/fpsyg.2018.01409

**Published:** 2018-08-15

**Authors:** Kenneth R. Paap, Regina Anders-Jefferson, Lauren Mason, Katerinne Alvarado, Brandon Zimiga

**Affiliations:** Department of Psychology, San Francisco State University, San Francisco, CA, United States

**Keywords:** bilingualism, inhibitory control, selective attention, visual search, ambiguous figures

## Abstract

A large sample (*N* = 141) of college students participated in both a conjunctive visual search task and an ambiguous figures task that have been used as tests of selective attention. Tests for effects of bilingualism on attentional control were conducted by both partitioning the participants into bilinguals and monolinguals and by treating bilingualism as a continuous variable, but there were no effects of bilingualism in any of the tests. Bayes factor analyses confirmed that the evidence substantially favored the null hypothesis. These new findings mesh with failures to replicate language-group differences in congruency-sequence effects, inhibition-of-return, and working memory capacity. The evidence that bilinguals are better than monolinguals at attentional control is equivocal at best.

## Introduction

Fluent bilinguals have acquired two lexicons and two grammars and must be able to select the intended words and rules as they switch back and forth between their two languages. This is usually viewed as non-trivial because both languages are coactivated during production and comprehension (see Paap, 2019 for a review). For example, the intention to say “gato" may coactivate “cat" in a Spanish-English bilingual. A common assumption (e.g., Blumenfeld and Marian, 2014) is that the competition from “cat" is usually resolved early by inhibiting the CAT representation within the lexicon. Furthermore, having nipped CAT in the bud the articulatory features for producing “cat" may not always emerge as a competitor that requires response inhibition. If the inhibitory control exercised at either the lexical or articulatory-response levels involves a general (domain-free) inhibitory-control mechanism and if this recruitment of general inhibitory control is functionally greater than the levels sustained by monolinguals in speaking a single language and in pursuing the myriad of goals required by everyday life, then bilingual advantages in inhibitory control would result. As we have repeatedly speculated (Paap and Greenberg, 2013; Paap et al., 2015; Paap, 2018, 2019) this logical chain can be broken at any link and consequently it should not be a surprise that the evidence for a bilingual advantage in inhibitory control is weak, at best.

The main purpose of this article is to consider Bialystok et al. (2009) revised hypothesis that bilingual advantages occur in attentional systems rather than in general inhibitory control.

The roots for this shift can readily be traced to the 2009 review by Bialystok et al. (2004) that opens the debate with a section headed *Inhibition or selection?* The authors point out that most (but not all) of the evidence taken to support the assumption that bilingual language control recruits a healthy dose of inhibitory control merely supports the less specific assumption that there is ubiquitous competition between the two languages that must be resolved by some conflict resolution mechanism: inhibition, selection, or some combination of mechanisms. Bialystok (2017) is convinced that “*Joint activation requires that there is a mechanism for language selection to assure that use of the target language proceeds fluently.*” Furthermore, “*the assumptions are that this mechanism is part of a domain-general process and that the constant engagement of this process for language selection fortifies it for other purposes, including non-verbal ones…*” p. 234. A key outcome of this analysis is that in the absence of additional qualifying assumptions regarding the nature of specific tasks, both the original hypothesis based on inhibition and the revised hypothesis based on selection make the same prediction (viz., a bilingual advantage) for non-verbal interference tasks such as the Simon, spatial Stroop, or flanker.

We will return to this issue in the discussion, but suffice to say that because the revised hypothesis, like the original, assumes that joint activation and competition involves some form of general-purpose control that is strengthened through practice (bilingual experience), the vast literature comparing bilinguals to monolinguals on tests requiring conflict resolution remain relevant to the strength and scope of bilingual advantages in cognitive control. That literature will be reviewed next followed by a review of studies using tasks that more distinctively focus on attentional control.

## Language Group Differences in Interference Control

### Average Effect Sizes

An early meta-analysis appeared to provide compelling evidence (*g* = 0.40) for bilingual advantages in cognition (Adescope et al., 2010). However, the analysis was very broad in scope and with hindsight very likely influenced by the file-drawer problem and publication bias. Direct evidence for these biases was provided by de Bruin et al. (2014). Both Hilchey et al. (2015) and Sanchez-Azanza et al. (2017), who used a bibliometric approach, speculated that the 2013 article by Paap and Greenberg may have been a turning point whereby challenges to the bilingual advantage hypothesis were more common than not, in part, because of a decrease in bias in the published literature. No doubt the steady drum beat of null results in large-scale studies (with highly proficient and balanced bilinguals and ages ranging from six to older adults) published by the Basque Center on Cognition, Brain, and Language (BCBL) also contributed to this shift (Antón et al., 2014, 2016; Duñabeitia et al., 2014).

More recent meta-analyses converge on the conclusion that significant bilingual advantages in inhibitory control are relatively rare (15% of all comparisons in Paap, 2018), that the average effect sizes are very small, and that there remains some amount of publication bias, which when taken into account, completely eliminates the effect. In Paap (2018) the mean advantage across 146 comparisons using interference scores derived from non-verbal interference tasks was +4.4 ms. If the 146 effect sizes are treated as a single sample the Bayes Factor (using the JZS prior and Rouder’s calculator) favoring the alternative is only 2.9.

A meta-analysis by Lehtonen et al. (2018) examined bilingual advantages across six domains of executive functioning (with very similar outcomes), but their analysis of inhibitory control is central to this discussion. Their meta-analysis used a wider definition of inhibitory control tasks and identified a more heterogeneous set of 212 effect sizes compared to Paap (2018). The Lehtonen et al. (2018) analysis was restricted to comparisons that were independent, yielded standardized effect sizes, and based on participants 18 years and older. In contrast, the Paap meta-analysis included participants 6 years and older. The mean effect size for inhibition in Lehtonen et al. (2018) was Hedge’s *g* = +0.11 [+0.05, +0.18], but when corrected for bias the mean was no longer significant, *g* = -0.02 [-0.12, +0.08]. The differences between the two meta-analyses are complementary and the fact that they converge on the same outcome leads to the conclusion that the evidence for a bilingual advantage in inhibitory control is extremely weak.

### Advantages in the Elderly?

The topic editors have called for a greater focus on possible developmental effects. In that regard Bialystok (2017) often characterizes the research on inhibitory control as showing more consistent bilingual advantages in older adults compared to younger adults. When the two “extraordinary” outliers^1^ of older adults from the Bialystok et al. (2004) study are excluded there are 19 other comparisons using non-verbal interference tasks in the Paap (2018) database of non-verbal interference tasks. Only two show significant bilingual advantages and the mean advantage of +9.7 ms has a 95% CI that straddles zero (CI: -0.4, +19.9). Seniors do not show consistent bilingual advantages.

### Advantages in School Children?

Similarly, there is a lore that bilingual advantages in inhibitory control occur consistently in children. In order to test this view the Paap (2018) database was searched for studies using children in the range of 6–15 years old. Only 3 of 30 comparisons produced significant bilingual advantages and the mean bilingual advantage was +2.2 ms (95% CI: -7.9, +12.2). School children do not show consistent bilingual advantages in these non-verbal interference tasks.

### Task Differences?

Another challenge to testing for bilingual advantages is that the interference scores derived from different non-verbal interference tasks often show weak and non-significant inter-task correlations (Paap and Sawi, 2014) and low test–retest reliability (Paap and Sawi, 2016). Even more disconcerting the arrows version of the flanker task does not correlate with original letter version (Salthouse, 2010). A more promising outcome was recently reported by Paap et al. (unpublished) in a study comparing four closely matched versions of the Simon, horizontal spatial-Stroop, vertical spatial-Stroop, and flanker tasks in that the interference scores from the first three showed moderate inter-task correlations (*r*’s ≈ 0.4). The flanker task did not significantly correlate with the Simon or spatial Stroop tasks suggesting that the nature of conflict resolution may depend on whether the conflict arises from two dimensions of the same stimulus or between adjacent but separate stimuli. The latter characterizes the flanker task because participants must select the relevant central arrow among the irrelevant flankers using visuospatial attention. Many theorists have suggested that conflict in the flanker task is resolved by spatially attending to the target stimulus (e.g., Magen and Cohen’s, 2007, dimension-action model). If spatial attention is construed as a filter or the upregulation of task relevant information then it clearly contrasts with inhibition. This interpretation of the flanker task is timely with respect to the bilingual advantage controversy given Bialystok’s (2017) reframing of the hypothesis from inhibition to attentional control. However, it must be noted that there were no bilingual advantages in inhibitory control in any of the four tasks reported by Paap et al. (unpublished).

## Language Group Differences in Other Tasks Requiring Attentional Control

Bialystok’s revised hypothesis assumes that when lexical entries in the two lexicons are co-activated that it is the disengagement of attention from the non-target language, not inhibition, that is the mechanism responsible for facilitating selection of the target language and the mechanism that creates bilingual advantages in domain general cognitive control. The evidence for this revised hypothesis has been drawn from the five tasks discussed in this section. The first two, conjunctive visual search and the ambiguous figures task are quite new to the bilingual advantage debate and will be the focus of the new studies reported below.

### Conjunctive Visual Search

In a test of the bilingual advantage in selective-attention hypothesis Friesen et al. (2014) reported that bilingual adults outperformed their monolingual counterparts in a conjunctive visual search task. Participants were instructed to decide as quickly and accurately as possible whether a specified target was present among an array of distractors. Displays including a target were designated as target-present trials whereas displays consisting only of distractors were designated as target-absent trials. Task difficulty was manipulated by search type: (target present vs. target absent), discriminability (low vs. high), and distractor set size (5, 15, 25). Latency was the primary dependent measure with faster RTs indicating better performance.

As expected significant bilingual advantages in search time occurred only in the conjunctive search condition that had low discriminability stimuli. For unexplained reasons, results only for the target-present trials were reported and analyzed. The significant group differences led Friesen et al. (2014) to conclude that bilingualism improves top-down selective attention in young adults. More specifically the extensive practice bilinguals receive at disengaging attention from the non-target language produces far transfer in the form of an enhanced ability to disengage attention from the distractors and more quickly find the target in the conjunctive visual search condition.

Given the difficulties in replicating studies showing bilingual advantages in EF it is perhaps no surprise that Ratiu et al. (2017) failed to find any bilingual advantages in conjunctive search across a series of three experiments that use eye movements to separate search time from decision time during conjunctive visual search. The study is data rich and if bilingualism confers advantages in selective attention a consistent difference should have been observed across the three experiments. The only reliable group difference was observed in Experiment 3 and that was a bilingual disadvantage in decision times. Ratiu et al. (2017) conclude that their results show no bilingual advantages in attentional guidance, response initiation or overall search performance.

Although the Ratiu et al. (2017) results are very consistent across all three of their experiments and test the same research question as Friesen et al. (2014) (viz., Are bilinguals better than monolinguals in conjunctive visual search under difficult conditions?), their materials and procedures were quite different. Thus, one purpose of the present study was to conduct a close replication of the critical conditions of the Friesen et al. (2014) search task. Another failure to replicate would deepen the skepticism that bilinguals show consistent advantages in selective attention, at least as reflected in performance during conjunctive visual search.

### The Ambiguous Figures Task

It has been proposed that the ambiguous figures task also provides a measure of selective attention (Chun-Fat-Yim et al., 2017). Young adult participants were presented with seven sequences of 11 drawings one at a time. The first drawing in each set was an unambiguous object that gradually changed into a different unambiguous object. Based on prior testing the sixth card was the most ambiguous. The instructions were to predict the alternative object using the fewest number of drawings. The series continued until a correct response was made or the participant reached the last figure. The dependent measure was the mean number of drawings it took to identify the alternative object. Lower scores presumably reflect better selective attention. Given that the bilingual group identified the alternative object earlier on in the series compared to the monolingual group, Chun-Fat-Yim et al. (2017) suggested that bilinguals were better able to disengage attention from the salient features consistent with the first interpretation to those consistent with the second and evolving interpretation. Although Chun-Fat-Yim et al. (2017) allow that the ability to disengage the focus of attention in order to selectively attend to new information “*involves EF*,” they emphasize that it is not equivalent to EF and “*is not defined by its components such as inhibition*” p. 371. A second purpose of the present study is to conduct a close replication of the Chun-Fat-Yim et al. (2017) study using the same ambiguous figures task.

### Congruency Sequence Effects

Grundy et al. (2017) further pursued the hypothesis that bilinguals are better than monolinguals at disengaging attention by comparing the magnitude of congruency sequence effects (CSEs). CSEs are robust context effects observed in many choice RT tasks that include both congruent and incongruent trials. Alternative names include the Gratton Effect (Gratton et al., 1992), sequential congruency effects, and conflict adaptation effects. The term CSE will be used descriptively to describe a specific outcome, namely, that the congruency effect is significantly smaller following incongruent trials than following congruent trials. In their first two experiments using a flanker task Grundy et al. (2017) observed no language-group differences in the magnitude of the simple flanker effect, but bilinguals did have significantly smaller CSEs compared to monolinguals. The smaller CSE was interpreted as reflecting *“….more rapid disengagement of attention and greater ability to refocus on the current trial”* p. 45. The findings are asserted to *“…provide insight into why some studies show bilingual advantages on executive control tasks and some do not”* p. 52.

Paap (2018) discusses several reasons why the Grundy et al. (2017) results and interpretation should be discounted. First, the results have consistently failed to replicate. See Table 3 of Paap (2018) for descriptive and inferential statistics associated with 10 failures to replicate across three different laboratories. Second, the Grundy et al. (2017) account does not mesh with Botvinick’s influential Conflict Adaptation Model which assumes that CSEs are the consequence of activating control plans for trial n based on the amount of conflict detected on trial n-1 rather than a potentially disruptive carryover effect from trial n-1 to n. Third, the assumption that smaller CSEs are good and are caused by a more rapid disengagement of attention and better ability to refocus on the present trial produces a contradiction. Grundy et al. (2017) reported null results (no group differences in the magnitude of the CSEs) in their Experiment 3 which they suggest is due to the relatively long response stimulus intervals *“during which all participants would have had sufficient time to disengage attention”* p. 51. But, this cannot be the case because the CSEs were equally robust for both groups. If CSEs are the product of carryover effects and if all participants had sufficient time to disengage attention, then all participants should have CSEs near zero. A related, but subtly different point is that CSE magnitudes are unrelated to overall task performance^2^ and, consequently, do not provide insights into the necessary and sufficient conditions for predicting bilingual advantages that matter in everyday life.

### Switch Costs

Grundy et al. (2017) point out that in cued switching tasks, when the task shifts from one dimension (e.g., sort on color) to another (e.g., sort on shape), participants must rapidly disengage from information that was relevant and refocus on information that was previously irrelevant. They cite Prior and MacWhinney (2010) and Prior and Gollan (2013) as showing that switch costs are smaller for bilinguals compared to monolinguals. These two early studies showing bilingual advantages are very difficult to replicate. For example, Paap et al. (2017) reported null effects in a large sample study using three-different switching tasks. More generally, Lehtonen et al.’s (2018) meta-analysis based on 77 comparisons across various types of switching tasks showed an average effect size of *g* = 0.15 [+0.06, +0.24] that disappeared when corrected for publication bias, *g* = 0.02 [-0.09, +0.14]. The results are no different when the analysis is restricted to the clearly non-verbal color-shape task with manual responses: Based on our current database of 16 articles^3^ and 25 such tests the mean bilingual advantage is 4.9 ms and the 95% confidence interval straddles zero, *t*(24) = 0.87, *p* = 0.39, CI[-7, +16]. The apparently robust bilingual advantage in switch costs reported in the seminal article by Prior and MacWhinney (2010) has turned out to be anomalous. If Grundy et al. (2017) are correct to characterize switch costs as a valid measure of the disengagement of attention, then the meta-analyses offer no support for the hypothesis of bilingual advantages in attentional control.

### Inhibition-of-Return (IOR)

In Posner’s cue-target paradigm (described in Klein, 2000) the interval between the rapid onset of a peripheral cue and a later target is varied. When the target appears in the cued location the typical finding is a brief period of facilitation followed by a longer period of inhibition known as inhibition of return (IOR). According to Klein (2000), the appearance of IOR is dependent on how quickly attention is endogenously disengaged from the cued location. Even though using “inhibition” as a marker of attentional control is somewhat ironic in the present context, it appears that the relative timing of IOR provides a fairly direct test of the hypothesis that bilinguals have learned how to rapidly disengage attention. Grundy et al. (2017) cite Mishra et al.’s (2012) report that high-proficiency bilinguals display IOR effects at earlier SOAs than low-proficiency bilinguals as support for this hypothesis. However, the Mishra et al. (2012) study did not include monolinguals. In a study that actually did compare bilinguals (*n* = 24) to monolinguals (*n* = 28) there were no group differences in the time course of IOR (Hernández et al., 2010). Furthermore, in a replication and extension of their earlier work Saint-Aubin et al. (2018) tested a large sample of English–French bilinguals and reported no effects of L2 proficiency on the IOR. Saint-Aubin et al. (2018) concluded that there is no reliable evidence that mastering a second language leads to faster or more potent disengagement of endogenous attention.

### Working Memory as Executive Attention

Bialystok (2017) asserts that working memory (WM) capacity, conceptualized not as storage space, but as the extent to which resources are available to control attention *“…is compatible with the evidence found across the life span for bilingualism-dependent plasticity”* p. 249. A recent meta-analysis by von Bastian et al. (2017) evaluated this conceptualization of EF for bilingual advantages. A set of 88 studies with 108 independent comparisons were included. The average effect size was *g* = +0.11 [+0.03, +0.19]. Considering the Bayes Factor associated with each effect size there was a high degree of heterogeneity, mostly null effects, and little evidence for the alternative hypothesis. Neither age (children, younger adults, older adults) nor task mode (verbal versus non-verbal) moderated the variability in effect sizes. Lehtonen et al. (2018) also examined the WM domain and their meta-analysis of 243 effect sizes yielded a mean effect size of *g* = +0.07 [0.00, +0.13] that shifted to a disadvantage when corrected for bias, *g* = -0.07 [-0.17, +0.03]. The Lehtonen et al. (2018) meta-analysis reinforces the conclusion of von Bastian et al. (2017) that the findings *“challenge executive-attention accounts of bilingual advantages.”*

## Materials and Methods

### Procedures

All participants completed the following activities in this order: (1) the conjunctive visual search task, (2) the Raven’s test, (3) the language background questionnaire, (4) demographic questions, (5) the ambiguous figures task and (6) the multilingual naming task (MINT) of productive vocabulary (Gollan et al., 2012).

### Participants

The 141 participants were San Francisco State University (SFSU) undergraduate students who participated for credit or extra credit in a psychology course. The protocol was approved by the SFSU Institutional Review Board. All subjects gave written informed consent in accordance with the Declaration of Helsinki. Participants were 18–29 years old. The language background questionnaire is the same as that used by Paap et al. (unpublished) and appears in the appendix to that article. The means for bilinguals and monolinguals on several background and language-use variables are shown in **Table 1**.

**Table 1:** Differences between bilinguals and monolinguals on demographics and language use.

	Bilinguals		Monolinguals					
Measure	*n*	Mean	*n*	Mean	Diff	SE	*t*	*p*
Age	79	22.3	44	21.4	0.9	0.48	+1.92	0.058
Ravens	77	8.2	44	9.1	-0.9	0.48	-1.89	0.061
SES	79	4.1	44	4.9	-0.8	0.23	-3.50	0.001
Most proficient language	79	6.4	44	6.5	-0.1	0.11	-0.71	0.479
English MINT	69	61.7	39	63.9	-2.2	0.70	-3.31	0.002
Second most proficient language	79	5.2	44	0.8	+4.4	0.24	18.09	<0.001
% of time use most proficient	79	65.4	44	98.6	-33.2	3.31	-10.0	<0.001
Language switches per day	79	3.5	44	0.7	+2.9	0.22	12.98	<0.001

*Diff, Group Difference; SE, standard error.*

The groups do not significantly differ on the Raven’s measure of general fluid intelligence, but the non-significant difference favors the monolinguals. The results of the *t*-tests reported later do not change if Raven’s scores are taken as a covariate. SES is a composite measure of mother’s education, father’s education, and family income. When we sample from the SFSU student population the monolinguals typically have a significantly higher degree of SES compared to the bilinguals. However, in this population the measures of SES are never significantly correlated with any of the measures of executive functioning and, indeed, those correlations are often near zero (Paap and Greenberg, 2013; Paap et al., 2014, 2017; Paap et al., unpublished). As reported in the results SES did not significantly correlate with the measures of selective attention. Variables that are uncorrelated with the dependent variable cannot be the cause of a null result that would otherwise show a bilingual advantage.

As shown in **Table 1** the bilinguals actively use two languages. On average their second most proficient language is self-rated as a 5.2 and a rating value of 5 was labeled “*Almost as good as a typical native speaker on both everyday topics and specialized topics I know about.*” They use their other language about one-third of the time. Their mean frequency of switching is 3.5 on a five-point scale where 3 is “*a couple of times a day*” and 4 is “*several times a day.*”

### Visual Search Task

The visual search task was modeled on that used by Friesen et al. (2014). Participants were instructed to search for a blue-triangle target and to press the “1” key if it was present and to press the “0” key if it was not. The visual arrays remained on the screen until a response was made. The next visual array appeared immediately after a response was made. The target randomly appeared in one of the 26 locations on the screen. Given that Friesen et al. (2014) reported a bilingual advantage only in the low discriminability conjunctive search condition, the feature-search and high discriminability conditions were omitted. Thus, search type (target present vs. target absent) and distractor set size (5, 15, 25) were manipulated. In conjunctive search, two features (e.g., shape and color) need to be identified in the target stimulus (e.g., blue triangle) in order to distinguish it from the distractor stimuli. The distractors were purple triangles and blue diamonds. The targets and distractors have low discriminability because purple is similar to blue and diamonds are similar to triangles. There were 24 target-present trials and 18 target-absent trials. There were six displays in each combination of number of distractors and positive versus negative trials. The 42 displays were presented in a different random order for each participant.

### Ambiguous Figures

In the ambiguous figures task participants were presented with seven sequences of 11 black-and-white line drawings. For each sequence the drawings were presented one at a time. The participant sat about 45 cm away from a Dell computer screen and each of the line drawings projected a visual angle of about 6.0^°^. In each set of figures, the first was an unambiguous object that morphed in discrete steps into a different unambiguous figure. Participants were shown the first figure and were prompted with the label that most observers readily see, for example, “most people see this drawing as a seal.” As each successive figure from the series was presented they were asked “Does it still look like a seal?” If the participant indicated that it no longer looked like the start object, they were asked to guess what it might be morphing into. The first dependent variable for this ambiguous figures task (AF1) was the trial number of the drawing that no longer looked like the start object. The sequence continued until the participant correctly identified the new object. The second dependent variable (AF2) was the trial number of the drawing that was correctly identified as the new object.

To illustrate the difference between the variables consider the following scenario. A participant is shown the first figure of the seal/horse set and told that most people see a seal. As second, third, and fourth figures are shown the participant continues to report seeing a seal, but when shown the fifth figure from the sequence she says it no longer looks like a seal. Her AF1 score for this set is therefore “5”. If her response to the follow-up question is that it now looks like a horse, then her AF2 score would also be “5.” However, if she does not guess the identity of the new object until she is shown the seventh figure, then her AF2 score would be “7.” If the participant was unable to correctly identify the new object after seeing the 11th and last figure in the sequence the AF2 score was assigned a value of 11.

Given the assumptions of Chun-Fat-Yim et al. (2017) higher scores signal poorer ability to disengage attention. All participants saw the sets in the following order: Seal/Horse, Old Man/Lady, Apple/Face, Rat/man, Lady/Sax Swan/Squirrel, Body/Face. Chun-Fat-Yim et al. (2017) did not prompt their participants with the label of the start object and used only the second dependent measure. During pilot testing we discovered that some participants did not correctly recognize the start object or saw it as a visually similar but different object. Although we were reluctant to deviate from Chun-Fat-Yim et al.’s (2017) procedure, the upside is that the two dependent variables may reflect two stages of selective attention: a disengagement of the salient features that promote the interpretation of the start object (AF1) versus an engagement of the salient features associated with the other object (AF2).

## Results

### Visual Search

Search times less than 200 ms or more than 2.5 standard deviations above the participant’s mean for each condition were removed as were incorrect responses. This was identical to the procedures used by Friesen et al. (2014) Trials consisting of a target with no distractors provide a measure of the speed of basic perceptual-motor processes. There was no difference between the groups on these trials, *t*(115) = -1.14, *p =* 0.257.

**FIGURE 1:**
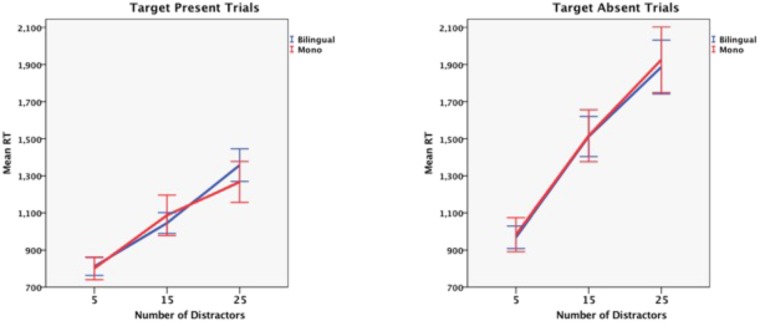
Mean search lime as a function of the number of distractors on target present **(left)** and target absent **(right)** trials. Errors bars are 95% confidence intervals.

Three-way mixed ANOVAs were performed separately on the RT and proportion correct (PC) data with Language Group (bilingual vs. monolingual) as a between-subjects factor and Trial Type (target present vs. target absent) and Number of Distractors (5, 15, 25) as repeated measures. The means and SEs in each condition are shown in **Table 2**. As expected, the RT analysis showed a significant main effect of Trial Type, *F*(1,115) = 140, *p* < 0.001 whereby it took longer to respond when no target was present and participants always had to search the entire display. Likewise the significant main effect of Number of Distractors, *F*(1,115) = 382, *p* < 0.001 confirmed that search times increase as the number of distractors increase. However, there was no significant main effect of Group, *F*(1,115) = 0.03, *p* = 0.854, nor was Group involved in any significant interactions. **Figure 1** shows the mean search time (for each group) as a function of the number of distractors for target-present and target-absent trials. Visual inspection confirms that there are no trends favoring bilingual advantages in search time.

**Table 2:** Means and standard deviations for reaction time and proportion correct for monolinguals and bilinguals in each condition defined by trial type and number of distractors.

	Positive trials Number of distractors				Negative trials Number of distractors		
	0	5	15	25	5	15	25
RT (*SD*)					
Bilingual	617 (8)	812 (24)	1046 (28)	1358 (44)	969 (30)	1512 (54)	1886 (73)
Monolingual	655 (30)	824 (37)	1097 (54)	1273 (54)	961 (50)	1482 (76)	1881 (96)
PC (*SD*)					
Bilingual	0.98 (0.06)	0.97 (0.09)	0.89 (0.15)	0.87 (0.14)	0.97 (0.07)	0.97 (0.08)	0.95 (0.09)
Monolingual	0.97 (0.09)	0.97 (0.07)	0.89 (0.12)	0.85 (0.16)	0.94 (0.17)	0.93 (0.17)	0.92 (0.18)

Given that Friesen et al. (2014) obtained bilingual advantages only in the low discriminability condition it is important to show that the low discriminability displays used in the present study produced comparable levels of difficulty. The means (estimated from Figure 3 of Friesen et al. (2014) and averaged across both language groups) for positive trials with 5, 15, and 25 distractors were about 980, 1300, and 1460 ms, respectively. Based on these values the slope of the best fitting straight line was 24 ms and this is very close to the 25 ms slope obtained for the positive trials in the present study. It seems that the conjunctive search conditions in the two studies are equally difficult.

Slope is arguably a purer measure of the ability to disengage and re-engage attention than overall search time. Consequently the slope for each participant across set sizes of 5, 15, and 25 distractors were computed for positive and negative trials separately (Pfister et al., 2013). Independent *t*-tests on these individual slopes compared language groups and showed no difference for either positive trials, *t*(119) = -0.55, *p* = 0.581, or negative trials, *t*(116) = 0.27, *p* = 0.781.

The mean proportion correct (PC) and SDs in each condition are shown in the bottom part of **Table 2**. The three-way mixed ANOVA showed no significant main effect of Language Group, *F*(1,115) = 2.274, *p* = 0.134; nor was Group involved in any significant interactions with Number of Distractors or Trial Type.

### Continuous Measures of Bilingualism

Rather than relying exclusively on categorizing participants as bilinguals, monolinguals, and undetermined; the entire sample can be used to examine the relationships between aspects of bilingualism (proficiency of the less dominant language, percentage of most used language, frequency of daily switching) and the measures of selective attention (RT, slope, and PC). These bivariate correlations are shown in **Table 3** and no aspect of bilingualism significantly predicts performance in the search task.

**Table 3:** Correlations between aspects of bilingualism and performance measures in the visual search task for all 127 participants.

	RT		PC		Slope	
Predictor	*r*	*p*	*r*	*p*	*r*	*p*
Proficiency of second most proficient language	+0.004	0.965	-0.041	0.646	-0.017	0.851
Percentage use of most used language	-0.062	0.486	+0.083	0.356	+0.148	0.096
Frequency of daily switches	-0.048	0.591	-0.037	0.681	-0.033	0.761

*RT, reaction time; PC, proportion correct; r, Pearson correlation; p, probability.*

### Ambiguous Figures

The first dependent variable, AF1, was the mean number of drawings examined before it no longer looks like the start object. The means for monolinguals (*n* = 43, *M* = 4.0) and bilinguals (*n* = 79, *M* = 4.1) did not significantly differ, *t*(120) = -0.247, *p* = 0.806. The second dependent variable, AF2, was the mean number of figures examined before correctly identifying the second object. Again, the means for monolinguals (*M* = 6.3) and bilinguals (*M* = 6.5) did not differ, *t*(120) = -0.735, *p* = 0.464, on this dependent variable either.

Despite the change in procedure that led to the addition of the first dependent variable, the overall mean of the ambiguous figure that yielded a correct identification of the new object was 6.4 in both studies. Our results offer no evidence of a bilingual advantage in the disengagement of attention. The continuous measures of bilingualism reported for the visual search task were also correlated with both dependent variables in the ambiguous figures task. All six correlations had magnitudes less than 0.09 and, consequently were not significant despite an N of 128.

### Bayes Factor Analyses

Bayes factor analyses calculate the ratio of probability of the null hypothesis given the data to the probability of the alternative given the data. The means and *t* values for the tests reported above were entered into Rouder’s Bayes Factor (BF) calculator (Rouder et al., 2009)^4^ using the default prior of *r* = 0.707. All of the Bayes factor analyses are greater than 3 which according to Jeffrey’s (1961) guidelines provide substantial evidence for the null hypothesis: overall search RT (4.5), overall search PC (4.7), slope on target trials (4.3), slope on no-target trials (4.7), AF1 (4.5), and AF2 (4.8).

### Correlations Between Measures

**Table 4** shows the within and between task correlations for the visual search and ambiguous figures tasks. The target only condition is the mean for the displays consisting of a single target with no distractors. Individual differences in the target only condition are likely to reflect differences in basic perceptual-motor processing. The correlations of the target only condition with the two slope measures are near zero and this is consistent with the assumption that search rate is independent of basic speed of processing. The correlation between the target-present and target-absent slopes is significant, but small, suggesting non-trivial differences in how the two types of displays are searched. This is consistent with the version of the guided search model developed by Chun and Wolfe (1996) that posits the setting of an activation threshold that terminates a non-exhaustive search more often for target absent trials than those where the target is present.

**Table 4:** Pearson correlations between specified measures from visual search and ambiguous figures task.

	Present slope	Absent slope	AF1	AF2
Target only	-0.05	+0.01	+0.20^∗^	+0.13
Target present slope		+0.21^∗^	-0.08	+0.07
Target absent slope			+0.04	+0.15
AF1				+0.67^∗∗^
AF2				1

*AF1, ambiguous figure 1 (no longer looks like the start object); AF2, ambiguous figure 2 (correct identification of the new object).*

Turning to the two dependent variables measured in the ambiguous figures task it is not surprising that they are highly correlated as no longer seeing a figure as the start object should facilitate being able to organize the features into a new object. Of primary interest is whether there are cross-task correlations that would support the possibility that both tasks are tapping into a shared attentional control mechanism. But, as evident in **Table 4** neither slope measure significantly correlates with either AF measure. There is a significant correlation between the target only RT and the first AF measure, but there is no obvious reason why general processing speed should be related to a judgment made under no time pressure.

## Discussion

The main empirical goal of this study was to conduct a close, but not exact, replication of two studies interpreted to support bilingual advantages in attentional control, particularly the ability to disengage attention. The conjunctive visual search task that yielded a bilingual advantage in Friesen et al. (2014) showed null results in the present experiment despite the fact that the studies produced nearly identical slopes of search time as a function of number of distractors. Furthermore, by examining slopes, target-absent trials, Bayes factors, and continuous measures of bilingualism the present study provided more tests of the hypothesis. Thus, the present study, together with the null results reported by Ratiu et al. (2017) seriously dampen the likelihood that bilingual advantages will consistently occur in search tasks.

The close replication of the Chun-Fat-Yim et al. (2017) study yielded overall means for identifying the new object that were identical in the two studies, but the present study showed no differences between bilinguals and monolinguals. The present study added a dependent variable (AF1, the drawing that no longer looks like the start object) that potentially separates attentional disengagement from re-engagement, but still no group differences were observed. One possible reason for the group differences reported by Chun-Fat-Yim et al. (2017) is that their bilinguals had higher maternal education, marginally higher fluid intelligence (*p* = 0.051), and a higher proportion of immigants.

### The Revised Hypothesis Revisited

Bialystok’s revised hypothesis is plausible and quite appealing, but before it can be rigorously tested it needs further specification. The looseness of the construct is reflected in the absence of a pater familias as in different articles and across different contexts the revised hypothesis is described in terms of executive attention, selective attention, or the disengagement of attention. Here we will introduce the term *attentional control* for a hypothetical construct that is presumed to be critical for bilingual language control. What is its essence? Are there any defining features or are there only characteristic features? If an important aspect of attentional control is the ability to focus on task relevant information and ignore irrelevant distracting information, then different types of selection are possible. In a flanker task a designated target object can be selected at the expense of the irrelevant object by spatially attending to the target. At least in theory, selection could also be the conflict resolution mechanism in a Simon task, but not via spatial attention because the task relevant information (e.g., color) and irrelevant information (e.g., location) are two attributes of the same stimulus. Neither of these types of selection seems to have much in common with selecting the lexical entry “gato” (or the entire Spanish lexicon) and leaving “cat” (or the entire English lexicon) behind when asked to name a picture of a domesticated feline in Spanish. The point here is that shifting the conflict-resolution mechanism from inhibition to attentional control doesn’t solve the problem of identifying the specific mechanism(s) used during bilingual language control and the degree to which the mechanisms are shared with non-verbal tasks.

In the absence of a more detailed proposal regarding the attentional control involved in bilingual language control, it is difficult to predict when bilingual advantages should occur and when they would be unlikely to occur. This allows the non-productive practice of attributing bilingual advantages to attentional control when differences occur and ignoring the null results. Are we foisting the results of the non-verbal interference tasks on Bialystok’s revised hypothesis? Beyond the logical argument drawn above consider that Bialystok (2017) includes the antisaccade, stop-signal, color-shape switching, and Simon as tasks that fall “broadly into a category of attention tasks” (p. 241). Furthermore, Bialystok suggests that the attentional system enhanced by bilingualism is similar in many respects to Posner’s “executive attention.” Yet executive attention is operationally defined in the seminal article by Fan et al. (2002) as the flanker interference effect (incongruent RT – congruent RT) in the attentional network task (ANT). Furthermore, Fan et al. (2002) state that executive control is defined as resolving conflict among responses.

To reiterate, if resolving the conflict between a bilingual’s two languages is the presumed cause of bilingual advantages and if this conflict-resolution mechanism recruits a general control ability, then bilingual advantages should occur in a wide array of non-verbal interference tasks. The only way to avoid this prediction is to make an additional *post hoc* assumption that only a subset of interference tasks use the general-purpose attentional control mechanism as a conflict resolution mechanism. Therefore, what is needed is a principled way to sort interference tasks into those where the conflict resolution mechanism is clearly attentional selection (and according to the revised hypothesis should show bilingual advantages) and those where conflict resolution relies on inhibition or some other task-specific mechanism (and consequently, according to the revised hypothesis should not show bilingual advantages). One step toward clarifying a construct of attentional control might use latent-variable analyses to determine if measures assumed to reflect attentional control all load on a common factor even if subsets are separable. If no such latent structure exists, then the hypothetical attentional-control construct may simply be chimerical.

## Conclusion

The review of the relevant prior literature showed that significant bilingual advantages in executive functioning (and especially the inhibitory control component) were relatively rare and that the average effect size was very small and plausibly due to file drawer and publication biases. Despite the exciting early reports of bilingual advantages, advantages in inhibitory control for bilinguals age six and older and for bilinguals who are older adults are more myth than reality. The proposal that bilingual advantages are rooted in attentional control rather than executive functioning is worthy of investigation, but the challenges are mounting rapidly as this revised hypothesis is tested in conjunctive visual search, the ambiguous figures task, CSEs, and IOR. Furthermore, to the extent that tasks such as the flanker or color-shape switching also recruit attentional control, these too should consistently produce bilingual advantages, not null results and effect sizes that straddle zero.

## Author Contributions

The selective attention studies formed part of a masters thesis completed by RA-J. All authors contributed to the design and conduct of the experiments. RA-J, LM, KA, and BZ coded the MINT and ambiguous figures recordings and contributed to the APS poster that reported the results of the experiments. RA-J and KP performed the data-analyses.

## Conflict of Interest Statement

The authors declare that the research was conducted in the absence of any commercial or financial relationships that could be construed as a potential conflict of interest.

## Footnotes

1 See Paap (2018) for the criterion used to identify outliers and a discussion of how strikingly anomalous these Simon effects were for older monolinguals.

2 When non-verbal interference tasks include an equal number of congruent and incongruent trials the CSE (the smaller congruency effect following an incongruent trial) is caused by a symmetrical speed-up on incongruent trials and slow-down on congruent trials (see **Figure 1** of Paap et al., 2016). The net result is that overall task performance remains unchanged and independent of the magnitude of the CSE.

3 The articles are listed in Table 2 of Paap (2018).

4 pcl.missouri.edu
